# Supplementary material for: Chimeric Pneumoviridae fusion proteins as immunogens to induce cross‐neutralizing antibody responses
Source: EMBO Mol Med. 2017 Dec 7;10(2):175–87. doi: 10.15252/emmm.201708078 (PMC5801496; doi:10.15252/emmm.201708078)
Supplement: Supplementary file 1 — Expanded View Figures PDF [file EMMM-10-175-s001.pdf]

Expanded View Figures

CODE	MUTANT	SEQUENCE	TRANSIENT EXPRESSION
		260 270 280	
	POST hRSV F	ELLSLINDMPITNDQKKLMSNNVQI	100
**F-400**	Post hRSV F / Site II hMPV F	ELLSLINDMPITAGQIKLMLENVQI	< 1
**F-401**	Post hRSV / Site II hMPV F	ELLSLINDMPTSAGQIKLMLENVQI	< 1
	POST hMPV F	ELARAVSNMPTSAGQIKLMLENRAM	
		230 240 250	
		420 430 440 450	
	POST hRSV F	VSCYGKTKCTASNKNRGIIKTFSNGCDYVSNKGVDT	100
**F-403**	Post hRSV F / SiteIV hMPV F	VSCYGKTKCTASNKNVGIIKQLNKGCDYVSNKGVDT	< 1
**F-404**	Post hRSV F / SiteIV hMPV F	VSCYGKTKCSIGSNRVGIIKQLNKGCDYVSNKGVDT	< 1
	POST hMPV F	VACYKGVSCSIGSNRVGIIKQLNKGCSYITNQDADT	
		380 390 400 410	
		260 270 280	
	PRE hRSV F	SELLSLINDMPITNDQKKLMSNNVQIVR	100
**F-407**	Pre hRSV F / Site II hMPV F	SELLSLINDMPITAGQIKLMLENVQIVR	4
**F-408**	Pre hRSV F / Site II hMPV F	SELLSLINDMPTSAGQIKLMLENVQIVR	2
	POST hMPV F	AELARAVSNMPTSAGQIKLMLENRAMVR	
		230 240 250	
		420 430 440 450	
	PRE hRSV F	VSCYGKTKCTASNKNRGIIKTFSNGCDYVSNKGVDTV	100
**F-410**	Pre RSV F / SiteIV hMPV F	VSCYGKTKCTASNKNVGIIKQLNKGCDYVSNKGVDTV	**68**
**F-411**	Pre RSV F / SiteIV hMPV F	VSCYGKTKCSIGSNRVGIIKQLNKGCDYVSNKGVDTV	**45**
**F-412**	Pre hRSV F / SiteIV hMPV F	VSCYKGVSCSIGSNRVGIIKQLNKGCDYVSNKGVDTV	23*
	POST hMPV F	VACYKGVSCSIGSNRVGIIKQLNKGCSYITNQDADTV	
		380 390 400 410 420	
		220 230 240 250	
	POST hMPV F	TDAELARAVSNMPTSAGQIKLMLENRAMVRR	100
**F-414**	Post hMPV F / Site II hRSV F	TDAELARAVSNMPTSNDQKKLMSNNRAMVRR	97
**F-415**	Post hMPV F / Site II hRSV F	TDAELARAVSNMPITNDQKKLMSNNRAMVRR	**96**
**F-416**	Post hMPV F / Site II hRSV F	TDAELLSLINDMPITNDQKKLMSNNRAMVRR	**10**
	POST hRSV F	TNSELLSLINDMPITNDQKKLMSNNVQIVRQ	
		250 260 270 280	
		380 390 400 410 420	
	POST hMPV F	LVACYKGVSCSIGSNRVGIIKQLNKGCSYITNQDADTVTI	100
**F-417**	Post hMPV F / Site IV hRSV F	LVACYKGVSCSIGSNRRGIIKTFSNGCSYITNQDADTVTI	8
	POST hRSV F	IVSCYGKTKCTASNKNRGIIKTFSNGCDYVSNKGVDTVSV	
		410 420 430 440 450	

Figure EV1. List of wild-type and chimeric proteins encoded in pRB21 plasmids.

The code of each chimeric protein is indicated in the left-hand column and distributed in groups. In each group, the name in the upper line denotes the wild-type protein used as backbone and the bottom line the protein from which antigenic sites were grafted in the chimeras. Numbers above and below sequences correspond to residues of the indicated F proteins. The first part of the chimera name refers to the source of the backbone and the second part to the grafted antigenic site. hRSV F and hMPV F sequences are colored red and blue, respectively. Residues shared by both sequences are colored purple. Transient expression levels were measured in culture supernatants by ELISA as indicated in Materials and Methods, using an anti-foldon mAb (common to all proteins) to capture the proteins that were developed with an anti-His mAb. Expression levels of chimeric proteins were normalized to that of the corresponding wild-type backbone protein, taken as 100%. Numbers shown in boldface indicate the chimeras that were incorporated to vaccinia virus recombinants. *Denotes a chimera that was not included in vaccinia virus for reasons explained in the text.

 © 2017 The Authors

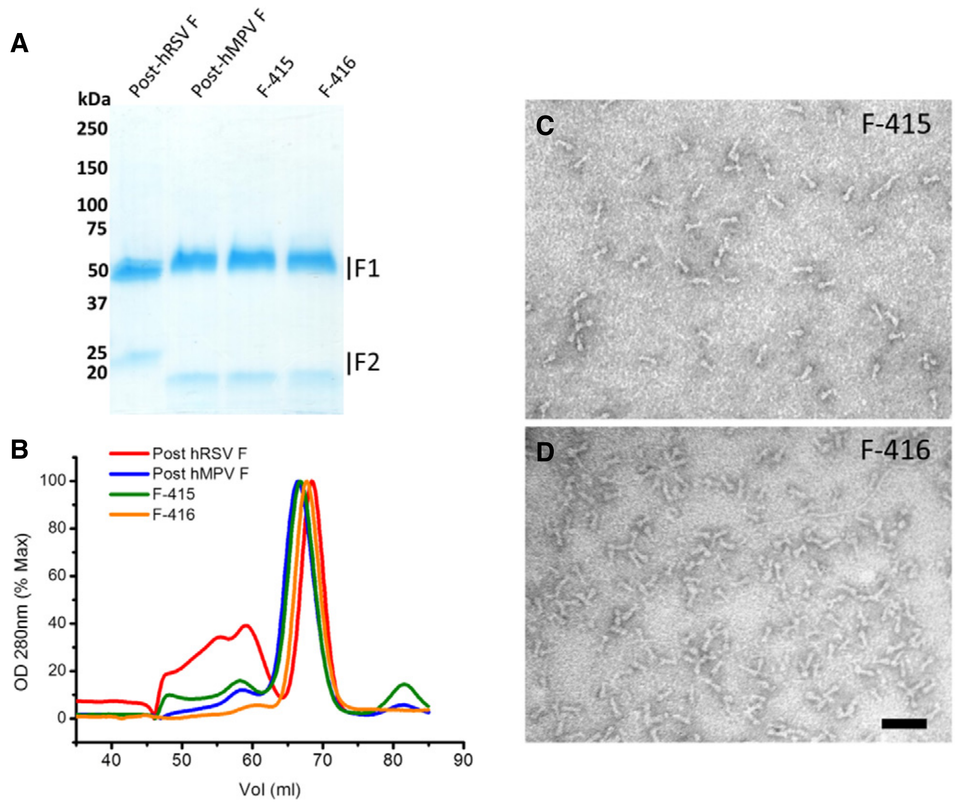

Figure EV2. Production and characterization of F-415 and F-416 chimeras.

A Coomassie-stained SDS–PAGE of the proteins indicated in each lane. Numbers on the left correspond to molecular mass markers (kilodalton, kDa). The position of F1 and F2 subunits is indicated on the right.

B Gel filtration chromatography profiles of the indicated proteins.

C, D Negative-stained EM images of purified F-415 (C) and F-416 (D). Scale bar, 50 nm.

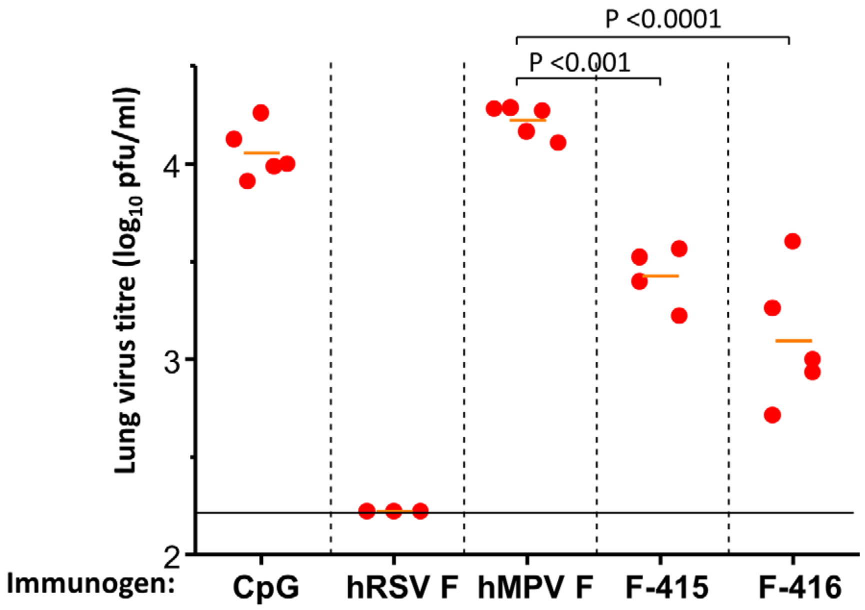

Figure EV3. Adoptive transfer protection of mice against a hRSV challenge.

Sera from groups of mice inoculated with the indicated immunogen and bled immediately before the challenge of Fig 4 were pooled together and diluted with an equal volume of PBS. 200 μl of the diluted pool was inoculated i.p. to each mouse 24 h before i.n. challenging with 4×10^6 pfu/mouse of hRSV (A2 strain) as indicated in Materials and Methods. Five days after challenge, mice were sacrificed and the amount of virus in lung extracts quantified by plaque assay. The number of mice per group was five except in the case of those receiving serum from mice inoculated with hRSV F (3 mice) and F-415 (4 mice) due to shortage of sera. Each dot represents an individual mouse. Short horizontal bars indicate mean values for each group. *P*-values were calculated as indicated under "Statistical Analysis". Only relevant *P*-values are shown. Differences were considered significant when $P < 0.05$. Long horizontal lines indicate detection limits.

© 2017 The Authors

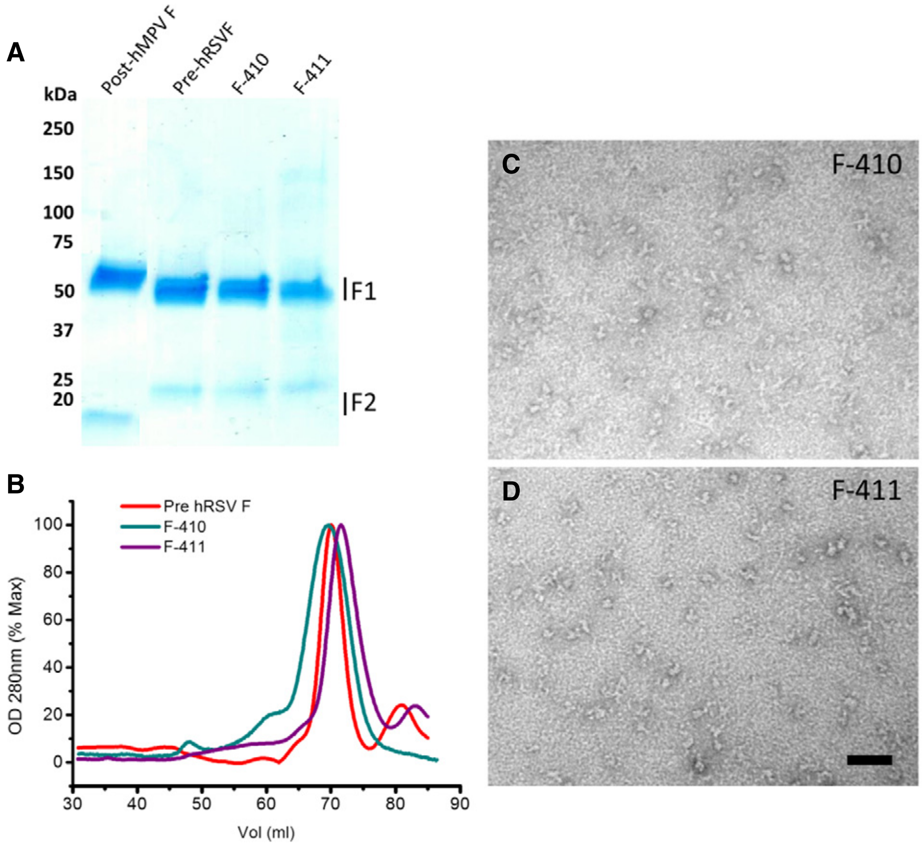

Figure EV4. Production and characterization of F-410 and F-411 chimeras.

A Coomassie-stained SDS–PAGE of the proteins indicated in each lane. Numbers on the left correspond to molecular mass markers (kilodalton, kDa). The position of F1 and F2 subunits is indicated on the right.

B Gel filtration chromatography profiles of the indicated proteins.

C, D Negative-stained EM image of purified F-410 and F-411, respectively. Scale bar, 50 nm.

 © 2017 The Authors

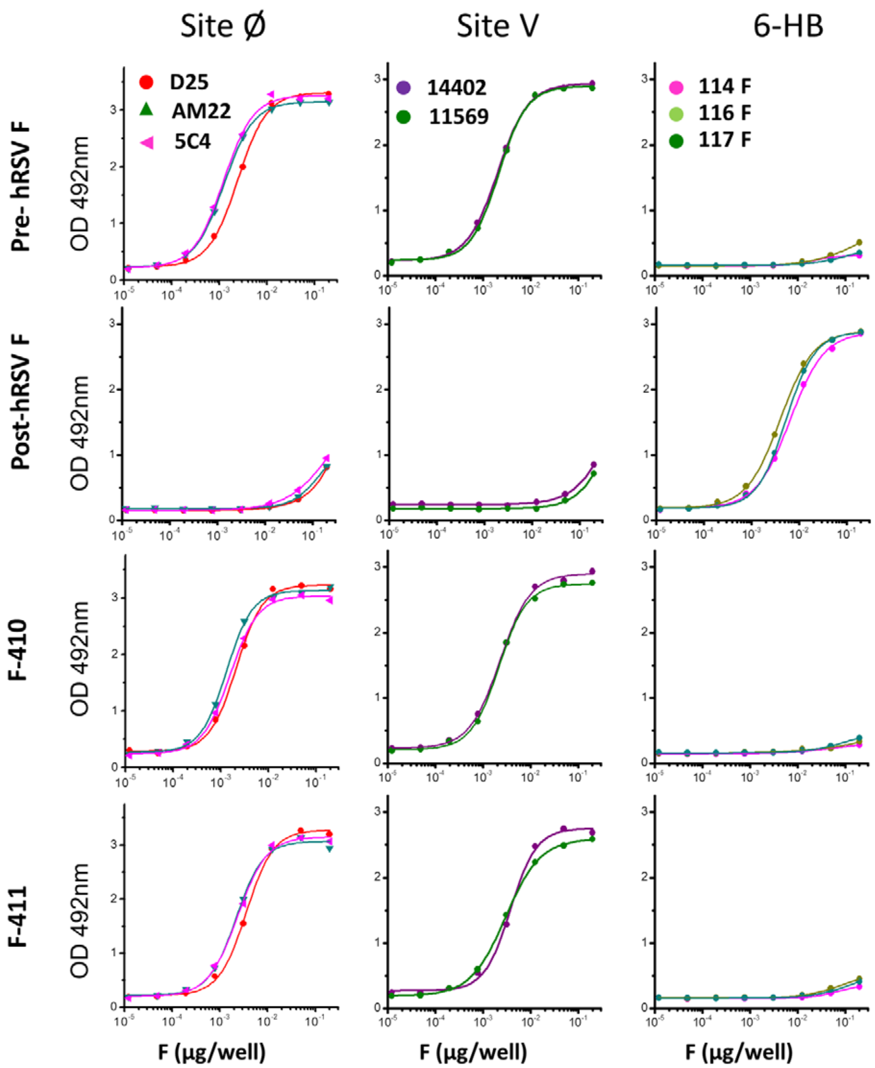

Figure EV5. Further antigenic characterization of the F-410 and F-411 proteins.

ELISA binding results of the proteins indicated on the left. Protein dilutions were captured with the mAbs indicated in the upper panels and revealed with an anti-His antibody, as described in Materials and Methods. D25, AM22, and 5C4 antibodies recognize epitopes of the hRSV F prefusion-specific antigenic site Ø (McLellan *et al*, 2013b). 14402 and 11569 antibodies recognize epitopes of the hRSV F prefusion-specific antigenic site V (Gilman *et al*, 2016). 114F, 116F, and 117F antibodies recognize epitopes of the 6-HB motif specific of postfusion hRSV F (Rodriguez *et al*, 2015).
